# Neural Efficiency and Attentional Instability in Gaming Disorder: A Task-Based Occipital EEG and Machine Learning Study

**DOI:** 10.3390/bioengineering13020152

**Published:** 2026-01-28

**Authors:** Riaz Muhammad, Ezekiel Edward Nettey-Oppong, Muhammad Usman, Saeed Ahmed Khan Abro, Toufique Ahmed Soomro, Ahmed Ali

**Affiliations:** 1 Department of Biomedical Engineering, Yonsei University, Wonju 26493, Republic of Korea; riaz@yonsei.ac.kr (R.M.); ezekieledward@yonsei.ac.kr (E.E.N.-O.); 2 Faculty of Engineering Sciences, Ghulam Ishaq Khan Institute of Engineering Sciences and Technology, Topi 23460, Pakistan; m.usman@giki.edu.pk; 3 Department of Electrical Engineering, Sukkur IBA University, Sukkur 65200, Pakistan; saeed.abro@iba-suk.edu.pk; 4 Artificial Intelligence and Cyber Futures Institute, Charles Sturt University, Bathurst, NSW 2795, Australia; 5 Biomedical & Instrument Engineering Department, College of Energy and Engineering, Abdullah Al-Salem University, Khalidiya 72303, Kuwait

**Keywords:** gaming disorder, task-based EEG, occipital cortex, machine learning, spectral slowing

## Abstract

Gaming Disorder (GD) is becoming more widely acknowledged as a behavioral addiction characterized by impaired control and functional impairment. While resting-state impairments are well understood, the neurophysiological dynamics during active gameplay remain underexplored. This study identified task-based occipital EEG biomarkers of GD and assessed their diagnostic utility. Occipital EEG (O1/O2) data from 30 participants (15 with GD, 15 controls) collected during active mobile gaming were used in this study. Spectral, temporal, and nonlinear complexity features were extracted. Feature relevance was ranked using Random Forest, and classification performance was evaluated using Leave-One-Subject-Out (LOSO) cross-validation to ensure subject-independent generalization across five models (Random Forest, KNN, SVM, Decision Tree, ANN). The GD group exhibited paradoxical “spectral slowing” during gameplay, characterized by increased Delta/Theta power and decreased Beta activity relative to controls. Beta variability was identified as a key biomarker, reflecting altered attentional stability, while elevated Alpha power suggested potential neural habituation or sensory gating. The Decision Tree classifier emerged as the most robust model, achieving a classification accuracy of 80.0%. Results suggest distinct neurophysiological patterns in GD, where increased low-frequency power may reflect automatized processing or “Neural Efficiency” despite active task engagement. These findings highlight the potential of occipital biomarkers as accessible and objective screening metrics for Gaming Disorder.

## 1. Introduction

Gaming disorder (GD) was formally recognized by the World Health Organization (WHO) in the 11th revision of the International Classification of Diseases (ICD-11) in 2018. It is characterized by impaired control over gaming, an increasing preference for gaming over other activities, and an ongoing engagement in gaming despite negative consequences [1,2]. This recognition marked a critical turning point in understanding behavioral addictions, placing gaming disorder alongside substance use disorders and pathological gambling in terms of clinical significance [3]. The disorder has shown a particularly high prevalence among adolescents, with recent meta-analyses estimating rates of 8.6% globally, some regions reporting rates as high as 11.7% [4]. These alarming statistics underscore the urgent need for objective, reliable, and early detection methods to facilitate timely intervention and treatment [3].

Traditional assessments for gaming disorders have mostly included self-report questionnaires and clinical interviews, such as Young’s Internet Addiction Test and several DSM-5-based instruments [5]. However, these subjective evaluations are limited by the inability to grasp the underlying neurophysiological mechanisms of addiction, lack of consistency across studies, and possible response bias. This constraint has motivated researchers to look into neuroimaging and neurophysiological techniques that could provide objective biomarkers for gaming disorder diagnosis and monitoring [6]. Due to its non-invasiveness, affordability, high temporal resolution, and capacity to record real-time brain activity patterns linked to addictive behaviors, electroencephalography (EEG) has become the most promising approach among these [7,8].

Studies utilizing EEG have revealed that individuals with gaming disorders have distinct neurophysiological rhythms from healthy controls [8,9]. Research has repeatedly found abnormalities in specific EEG frequency bands where individuals with gaming disorder frequently showed increased theta power and decreased beta power, particularly in frontal and parietal regions. Furthermore, increased intrahemispheric gamma coherence has been identified as a fundamental neurophysiological component of gaming disorder, differentiating it from other types of behavioral disorders, such as alcohol use disorder. Event-related potential (ERP) studies have revealed variations in key cognitive processing components, including decreased P300 amplitudes during auditory oddball tests and altered N2 components during tasks that require inhibitory control. These anomalies reveal underlying mechanisms in decision-making, attention, and response inhibition [10,11]. These neurophysiological shifts correspond to the severity of gaming disorder symptoms and possess similarities with neural patterns found in substance use disorders, supporting the idea that gaming disorder is a behavioral addiction with specific brain-level manifestations. Spectral slowing, defined as a relative increase in low-frequency EEG activity (delta and theta bands) accompanied by a reduction in higher-frequency components, has been reported as a neurophysiological marker in various addictive and compulsive behaviors [12,13].

The integration of deep learning (DL) and machine learning (ML) methods with EEG signals has transformed the prediction and identification of gaming disorders. Recent studies have demonstrated that sophisticated machine learning algorithms, such as Random Forests (RFs), Support Vector Machines (SVMs), Convolutional Neural Networks (CNNs), and Long Short-Term Memory (LSTM) networks, can differentiate between people with gaming disorders and healthy controls with classification accuracy ranging from 85% to 93% [5]. Notably, emerging CNN-based approaches offer scalable alternatives to traditional ICA-based artifact decomposition, enabling faster preprocessing of large EEG datasets without compromising signal quality [14]. These models typically employ advanced feature extraction approaches, including wavelet transforms, Power Spectral Density (PSD), time–frequency analysis using Fast Fourier Transform (FFT), and functional connectivity metrics such as EEG coherence [15]. However, these high-performance models often rely on complex, computationally intensive deep learning architectures that function as “black boxes,” lacking the interpretability required for clinical adoption. Furthermore, a multimodal technique usually combines EEG with additional physiological signals such as photoplethysmogram (PPG) and electrooculogram (EOG). It could also integrate EEG with other behavioral data and neuroimaging modalities, such as Functional near-infrared spectroscopy (fNIRS) and Positron emission tomography (PET). These multimodal systems are more effective than single-modality techniques [6,15].

Despite these advancements, there are still significant barriers to the clinical application of EEG-based gaming disorder detection and identification. Recent machine learning studies have demonstrated the discriminative potential of specific EEG frequency bands, including gamma-band activity, for cognitive state classification [16]. When compared to fMRI, EEG has lower spatial resolution and is more vulnerable to aberrations from eye movements and muscle activity [17]. Furthermore, the absence of standardized EEG recording protocols, inconsistent feature extraction methodologies among studies, and low generalizability of models trained on small datasets all impede the wider adoption of these techniques in clinical settings. Specifically, the occipital visual cortex (O1/O2) remains under-investigated despite its central role in processing the intense, reward-linked visual stimuli that drive the “compulsion loop” in video games [18,19]. Unique sensory-gating mechanisms not seen in frontal or resting-state recordings may be revealed by directly capturing neural dynamics from the visual processing center during active gameplay.

**Figure 1:**
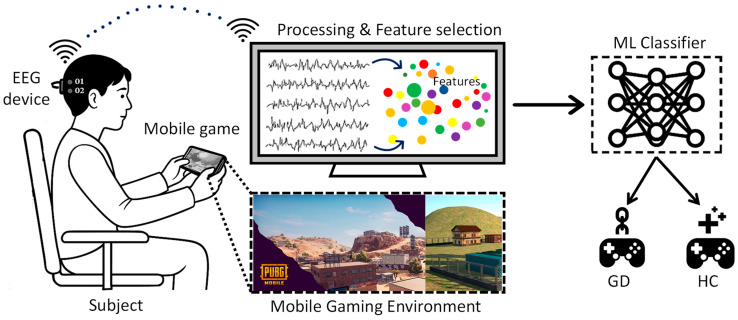
Overall experimental setup for wireless EEG-based analysis of mobile gaming behavior. A participant wearing a wireless EEG headset (OpenBCI Ultracortex Mark IV) plays a mobile game while neural signals are acquired using two occipital electrodes (O1 and O2). EEG data are transmitted to a processing unit, where raw EEG epochs undergo feature extraction and visualization before being fed into a machine-learning classifier for behavioral state prediction. The inset shows representative game environments and classification labels: GD (Gaming Disorder) and HC (Healthy Controls), indicating the two behavioral categories used in the study.

Developing EEG-based assessment frameworks that are low-cost, wireless, and suitable for real-world deployment remains a key challenge in bioengineering research on behavioral addictions. In this study, we develop a data-driven machine learning framework to predict Gaming Disorder (GD) from task-based occipital EEG in order to provide an objective early screening tool that supports questionnaire-based diagnosis. We focus on physiologically interpretable spectral, temporal, and nonlinear features extracted from O1/O2 signals and rigorously compare different conventional classifiers to find the most reliable ML model for GD detection. By isolating robust occipital EEG biomarkers that distinguish gamers with GD from healthy controls, this work seeks to improve diagnostic precision and to clarify the neurophysiological mechanisms of behavioral addiction, thereby supporting the development of more targeted and personalized intervention strategies. An overview of the experimental procedure is given in Figure 1, which shows how EEG is recorded while playing mobile games, how neural characteristics are extracted from the O1 and O2 channels, and how machine learning classifiers are then used to distinguish addicted gamers from healthy controls.

As gaming disorders continue to rise among teenagers and young adults in an increasingly digital world, the need for reliable early detection tools has become more urgent. Developing accessible and interpretable EEG-based screening approaches, therefore, represents an important step toward enabling objective assessment in real-world and point-of-care settings. In this study, we examine task-based occipital EEG dynamics during active gameplay using an exploratory framework to identify whether low-frequency spectral alterations associated with gaming disorder may reflect adaptive or automated visual processing patterns rather than definitive neurophysiological markers.

## 2. Materials and Methods

### 2.1. Participants and Experimental Setup

EEG data were obtained from 30 healthy adult volunteers (age range: 21–27 years; mean ± SD: 24.0 ± 3.0 years). All participants reported having normal or corrected-to-normal vision, were right-handed, and gave written informed consent in compliance with institutional ethical standards. Participants were categorized into Gaming Disorder (GD) and Healthy Controls (HC) groups based on the Comprehensive Scale for Assessing Gaming Behavior (CSG, 2010), a validated 20-item assessment instrument widely used to quantify problematic gaming tendencies across behavioral and psychological dimensions [20,21]. The Gaming Disorder (GD) group (*n* = 15) comprised participants with ≥15 h daily gaming engagement and high Maladaptive Game Use Scale (MGUS) scores (≥3 of 7 subfactors scoring ≥ 6), indicating problematic gaming across dimensions including tolerance, withdrawal, excessive time, loss of control, continued use despite problems, deception, and escape. The Healthy Control (HC) group (*n* = 15) comprised participants with <1 h of daily gaming and low Adaptive Game Use Scale (AGUS) scores, representing normative gaming patterns without problematic symptoms.

During data acquisition, subjects engaged in a 10-min continuous mobile action game PUBG Mobile (PlayerUnknown’s Battlegrounds). This specific title was selected because it involves fast visuomotor coordination, high sensory engagement, and reward-driven repetition, characteristics that have been previously reported to modulate occipital EEG rhythms and contribute to addictive gaming patterns.

#### 2.1.1. CSG Classification and Borderline Case Handling

The CSG employs a bidimensional classification system distinguishing adaptive and maladaptive gaming profiles. For this study, participants were assigned to GD or HC groups based on MGUS scores in conjunction with daily gaming hours. Borderline cases were defined as MGUS scores between the 40th and 60th percentiles of the total score distribution. Out of the initial screening pool (*N* = 42), 12 individuals met borderline criteria and were excluded from analysis to ensure clear phenotypic separation. The final cohort comprised 30 participants with clearly delineated GD (*n* = 15) and HC (*n* = 15) phenotypes.

#### 2.1.2. Recruitment Procedure and Verification

Participants were recruited through university student email lists and laboratory announcements posted on campus bulletin boards, targeting undergraduate and graduate students aged 21–27 years. Daily gaming hours were self-reported via a structured interview during initial screening and verified against participants’ own app usage statistics (iOS Screen Time, Google Play data) when available.

### 2.2. EEG Data Acquisition

#### 2.2.1. Recording Device

A customized OpenBCI Ultracortex Mark IV headset with dry EEG comb electrodes was used to record EEG signals. The device collected data at 256 Hz, which is sufficient for resting-state and task-related EEG research and surpasses the minimal Nyquist criterion for capturing oscillations up to the gamma frequency range.

Dry electrodes were chosen to provide sufficient signal quality for spectral and temporal feature extraction, while ensuring quick setup, minimizing preparation work, and preserving participant comfort throughout the gaming activity. Because of its open-source architecture, portability, and proven performance when compared to clinical EEG systems, the OpenBCI platform is increasingly popular in neuroscientific research.

#### 2.2.2. Electrode Placement

**Figure 2:**
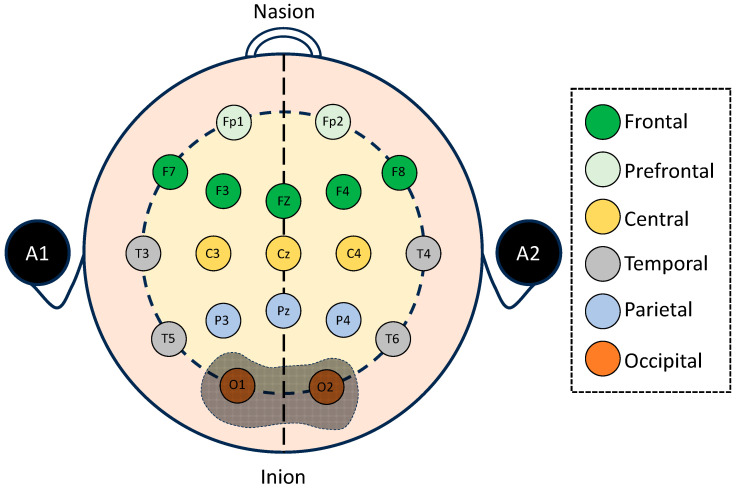
Schematic top-view layout of the OpenBCI Ultracortex Mark IV electrode montage, highlighting the 10–20 system positions. Background and electrode colors denote anatomical regions. The occipital region (O1 and O2), emphasized in brown shading, represents the primary locations used for EEG acquisition in this study. Frontal (green), frontopolar (light green), central (yellow), temporal (gray), parietal (blue), and midline (teal) electrodes are shown for anatomical reference, along with the left and right earlobe reference points (A1, A2). This visualization illustrates the spatial context of the recorded EEG channels analyzed for neural activity related to gaming disorder.

Two monopolar electrodes were positioned at the O1 and O2 sites according to the international 10–20 EEG system (Figure 2). The selection of these occipital locations was intentional because the occipital cortex plays a central role in visual processing, attentional allocation, and sensory integration. These neurocognitive domains are highly engaged during fast-paced and visually rich gaming tasks. Prior research has shown that occipital theta, alpha, and beta oscillations undergo measurable alterations in individuals with behavioral addictions, such as Internet Gaming Disorder, as well as in substance-related dependence and other technology-driven compulsive behaviors.

The main location of visual sensory processing is the occipital cortex. Neural efficiency and visual sensory gating during high-demand tasks can be evaluated by examining O1/O2 dynamics. Prior EEG studies have shown that occipital oscillations—particularly in the delta, theta, alpha, and beta bands—are sensitive to attentional load, sensory gating, and habitual visual processing, all of which are highly relevant to gaming disorder pathology [22,23].

#### 2.2.3. Recording Conditions

Recordings were made while each participant played a 10-min PUBG (PlayerUnknown’s Battlegrounds) game on a mobile device. PUBG was selected because it stimulates reward-driven engagement, fast perceptual-motor responses, and constant visual attention, all of which have been linked to altered brain processes in GD gamers and drug users. Participants were instructed to refrain from unnecessary blinking and muscular contractions while playing to minimize artifacts. PUBG Mobile (Battle Royale Mode) was played on a standardized iPhone 13 (30 cm viewing distance) with fixed device settings across all participants: Medium touchscreen sensitivity, balanced graphics (1080p, 60 fps), 70% audio volume. A 5-min familiarization session preceded the EEG recording to acclimate participants to the controls. During the 10-min recording, all participants maintained continuous active gameplay without pauses or interruptions; engagement was verified post-hoc via in-game action logs (character movement, combat interactions). All recordings occurred in a dimly lit room with standardized temperature (21–23 °C), seating, and lighting to ensure comparable conditions. While participants were instructed to minimize head movement, the nature of handheld mobile gaming inevitably involves minor postural adjustments and digital manipulations. These are acknowledged as ecological characteristics of the naturalistic gaming task.

### 2.3. Data Preprocessing

**Figure 3:**
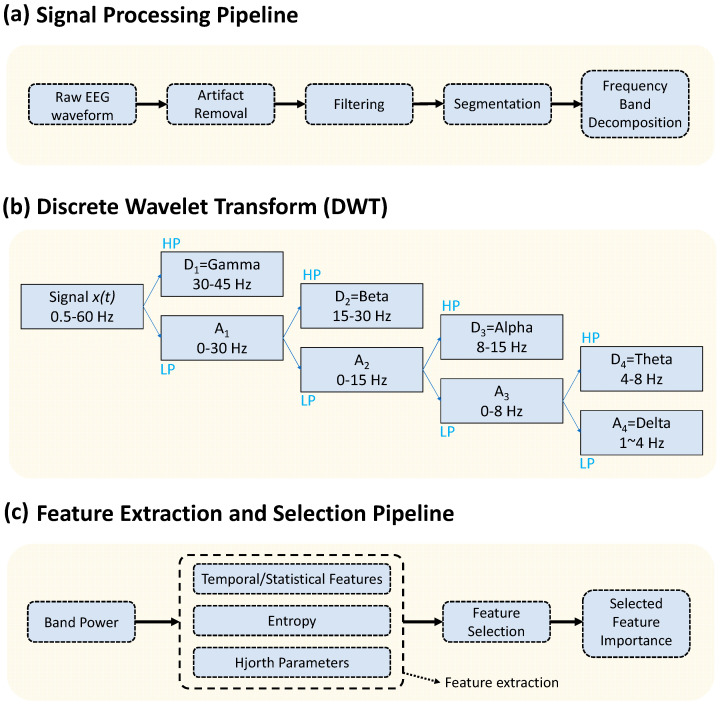
Overview of the EEG signal processing, wavelet-based frequency decomposition, and feature extraction pipeline. (**a**) illustrates the signal processing workflow, beginning with raw EEG acquisition, followed by artifact removal, filtering, segmentation, and subsequent frequency-band decomposition. (**b**) presents the multilevel Discrete Wavelet Transform (DWT) procedure used to derive band-specific components (Gamma, Beta, Alpha, Theta, Delta) from the original 0.5–60 Hz EEG signal. (**c**) summarizes the feature extraction and selection pipeline, in which band power, temporal/statistical descriptors, entropy measures, and Hjorth parameters are computed and passed through a feature selection stage to obtain the final feature set used for classification.

To ensure optimal signal quality and eliminate noise sources frequently found in single-channel and low-density EEG systems, the EEG recordings from channels O1 and O2 were preprocessed (Figure 3a). All preprocessing procedures were carried out using EEGLAB, MNE-Python, and internal scripts in accordance with accepted standards for biomedical signal cleaning.

#### 2.3.1. Filtering

Raw EEG signals were subjected to a multi-stage filtering strategy to preserve physiologically meaningful brain oscillations while eliminating noise. EEG signals were acquired using the OpenBCI system, which applies an online bandpass filter (0.5–60 Hz) during data acquisition. Offline preprocessing included the application of a single-notch filter at 50 Hz to suppress power-line interference corresponding to the local mains frequency [24]. The 50 Hz notch was applied before the final low-pass filter, which defined the effective analysis bandwidth (0.5–50 Hz) using a 4th-order zero-phase Butterworth filter. As commonly observed in mobile dry-electrode EEG recordings, a narrow residual spectral peak near 50 Hz was occasionally visible in the power spectra due to nonstationary line-noise coupling and filter roll-off characteristics. The choice of a 4th-order zero-phase Butterworth filter was informed by recent systematic evaluations of EEG power estimation pipelines, which demonstrated that forward–backward IIR Butterworth filtering provides an effective balance between artifact attenuation and preservation of physiologically meaningful oscillatory activity compared with alternative FIR and IIR designs [25]. To ensure robust high-frequency feature extraction, gamma-band analyses were restricted to the 30–45 Hz range, fully excluding the line-noise frequency. Post hoc frequency masking refers to the explicit exclusion of spectral bins at and around 50 Hz from power and entropy calculations. Because gamma features were computed well below the notch frequency and low-pass cutoff, contamination from line noise or filter-induced artifacts is minimized.

#### 2.3.2. Artifact Removal and Signal Segmentation

EEG recordings were preprocessed to minimize non-neural artifacts caused by movement, muscle activity, and electrode instability. The first 20 s of each recording were discarded to allow electrode-skin impedance stabilization, and the final 20 s were removed to avoid edge artifacts introduced by the digital filtering. This dual-boundary approach was selected based on OpenBCI Ultracortex Mark IV technical specifications and established protocols in dry EEG literature, which indicate that dry electrodes typically require 10–30 s for impedance stabilization and baseline shift settling [26,27,28]. The 20-s margin at each boundary represents a conservative approach, consistent with prior task-based EEG studies employing dry electrode systems. This uniform discarding protocol was applied across all 30 participants and sessions to ensure standardized preprocessing, resulting in 560 s of analyzable EEG data per participant.

To address low-frequency artifact concerns, continuous EEG was high-pass-filtered at 0.5 Hz (4th-order Butterworth, transition band 0.1–0.5 Hz) before artifact rejection. High-pass filtering removes DC drift and slow movement-related artifacts (0–0.5 Hz), which amplitude-based thresholds alone cannot effectively detect [29]. After high-pass filtering, the ±100 µV peak-to-peak amplitude criterion was applied to detect and reject remaining high-frequency muscle and movement artifacts [24]. Artifact detection was performed on high-pass-filtered data (0.5 Hz) to avoid drift-driven threshold violations; therefore, the amplitude criterion reflects post-filtered signal excursions rather than raw voltage. This two-stage approach mitigates major low-frequency drift and high-amplitude non-neural artifacts across frequency bands, within the constraints of a low-density dry-electrode montage. Manual visual inspection in EEGLAB was applied only as a confirmatory step due to the low-density two-channel montage, which precludes spatial decomposition methods such as Independent Component Analysis. Recent advances propose CNN-driven alternatives to traditional ICA decomposition. CNN-based ICA alternatives explicitly model spatial source separation, which is critical for disentangling ocular and muscle artifacts in multi-channel EEG [14]. Because the present study used a two-channel occipital montage, spatial decomposition was not identifiable, motivating the use of amplitude-based rejection instead. Visual review focused on verifying the removal of gross motor and muscle-related artifacts and on ensuring temporal consistency of artifact patterns across both occipital channels. To evaluate the robustness of low-frequency findings, sensitivity analyses were conducted using alternative amplitude thresholds (±75 µV and ±125 µV).

To evaluate potential bias introduced by the concatenation of artifact-free EEG segments, we performed an additional segment-wise feature extraction analysis. For each subject, spectral features were computed independently for each artifact-free 10-s segment, yielding (50 ± 6) segments per subject. A 10-s segment length was selected to ensure stable estimation of low-frequency (delta/theta) spectral power while maintaining sufficient temporal resolution for artifact rejection. Subject-level means were then calculated by averaging segment-level features within each subject, and group comparisons were conducted on subject-level aggregated features.

Once artifact-contaminated segments were identified and removed, the remaining artifact-free epochs were concatenated to form a continuous cleaned signal for subsequent analysis. All spectral, temporal, statistical, and entropy features (band power, PSD metrics, Hjorth parameters, Shannon entropy) were computed on the full-length cleaned band-limited signals rather than on individual epochs. This approach preserves the temporal continuity and sustained neurophysiological dynamics of the 10-min gameplay task, capturing global EEG patterns across the entire recording session.

### 2.4. Frequency Band Decomposition

#### 2.4.1. Discrete Wavelet Transform (DWT)

The EEG recordings from channels O1 and O2 were decomposed into physiologically meaningful frequency bands using the Discrete Wavelet Transform (DWT) (Figure 3b). DWT is widely used for decomposing EEG signals into multiple frequency bands, facilitating detailed time–frequency analysis. In this study, the Daubechies-4 (db4) wavelet was selected as the mother wavelet because of its compact support, orthogonality, and similarity to the EEG waveform morphology. The db4 wavelet is widely used in EEG analysis due to its ability to preserve transient components, minimize edge distortion, and provide stable reconstruction of neural oscillations. The db4 wavelet includes four scaling and wavelet coefficients that enable efficient multi-stage signal decomposition. The primary concept of DWT is to iteratively decompose the EEG signal input into a series of smaller localized waveforms through successive application of low-pass (LP) and high-pass (HP) filtering, followed by downsampling. This multiresolution approach converts the EEG signal into approximation and detail coefficients at several levels, which correspond to distinct frequency bands.

At each decomposition level, the input signal *x*(*t*) is convolved with a scaling function *ϕ*(*t*) (low-pass filter) and a wavelet function *Ψ*(*t*) (high-pass filter), yielding approximation coefficients *A_j_* and detail coefficients *D_j_*:

(1) $$A_{j}\left(k\right)=\underset{n}{\sum }x\left(n\right)\;h\left(2k-n\right)$$

(2) $$D_{j}\left(k\right)=\underset{n}{\sum }x\left(n\right)\;g\left(2k-n\right)$$

where *h* and *g* denote the low-pass and high-pass filter coefficients associated with db4. Successive application of these filters produces a hierarchical representation in which lower decomposition levels capture higher frequencies and higher levels capture lower frequencies. Using the sampling frequency of the dataset, each DWT node was mapped to one of the standard EEG bands. Detailed coefficients from the first level corresponded to the Gamma band, while subsequent detail coefficients represented Beta, Alpha, and Theta activity. The final approximation coefficients represented the Delta band. A complete illustration of the DWT pipeline used in this study is shown in Figure 3b.

#### 2.4.2. Extraction of Band-Limited O1 and O2 Signals

Following the DWT, band-limited EEG signals were reconstructed for each frequency band at both electrode sites (O1 and O2). This produced five time domain signals per channel corresponding to Delta (0–4 Hz), Theta (4–8 Hz), Alpha (8–15 Hz), Beta (15–30 Hz), and Gamma (30–45 Hz) activity. Different neurophysiological processes are represented by these oscillatory components. High cortical inhibition and slow-wave synchronization are linked to delta activity. Theta rhythms reflect cognitive control and attention processing. Occipital idling and visual disengagement are associated with alpha oscillations. Gamma activity indicates quick local processing, perceptual binding, and attentional burden, while beta activity indicates sensorimotor activation and increased cognitive engagement.

### 2.5. Feature Extraction

The band-limited EEG data obtained from the O1 and O2 channels were used to extract a wide range of features (Figure 3c). The goal was to capture the temporal, spectral, statistical, functional, and nonlinear aspects of the signals that could differ between GD and HC individuals. For every channel and frequency band (Delta, Theta, Alpha, Beta, and Gamma), each feature was calculated independently. Recent machine-learning studies on cognitive state classification demonstrate that gamma-band features in the 30–45 Hz range provide superior discriminative power [16]. Accordingly, Gamma band features were restricted to the 30–45 Hz frequency range to avoid overlap with line-noise contamination and its harmonics. When necessary, composite features (such as O1 + O2) were also produced. The feature categories were chosen based on previous EEG classification studies in cognitive impairment, addiction, and psychiatric illnesses, where band power, PSD-derived indices, entropy measurements, Hjorth parameters, and coherence all have shown discriminative potential.

#### 2.5.1. Band Power Features

Band power features were computed from the DWT-reconstructed signals in each band. Absolute band power was calculated as the squared magnitude of the signal:

(3) $$P_{abs}=\overset{N}{\underset{n=1}{\sum }}x{\left(n\right)}^{2}$$

where *x*(*n*) is the amplitude of the EEG signal at sample *n*, *N* is the total number of samples, and *P_abs_* is the absolute power of the specified EEG band of interest.

Relative band power (*P_rel_*) was computed as the ratio of power in a specific band to the sum of powers of all five bands:

(4) $$P_{rel}=\frac{P_{band}}{\sum_{i=\Delta ,\theta ,A,B,\Gamma }P_{i}}$$

where *P_i_* is the absolute power of each EEG band (Delta Δ, Theta θ, Alpha A, Beta B, Gamma Γ) for the same channel and time segment.

Band power was obtained for O1, O2, and the combined occipital region (O1 + O2). Band-limited power was computed from the band-pass-filtered signal as mean squared amplitude (µV^2^), which provides a time-domain estimate of average power in each frequency band. These characteristics, which are frequently employed in GD-related EEG research, indicate the intensity of oscillatory activity in the conventional EEG frequency ranges.

#### 2.5.2. PSD-Based Features

The Power Spectral Density (PSD) of the EEG signals was estimated by deriving spectral features using the Welch method, which uses a Hamming window with 50% overlap to minimize spectral leakage and volatility. As a marker for the dominant oscillation rate, the peak frequency was found to be the frequency component that contributed the most power within each band. Certain band power ratios, including the Theta/Alpha, Theta/Beta, and Beta/Alpha ratios, which are commonly used markers of arousal, attention, and cognitive load, were calculated to evaluate the balance between low-frequency and high-frequency activity.

#### 2.5.3. Temporal and Statistical Features

Statistical moments were computed for each decomposed sub-band signal to capture the time-domain distribution properties of the EEG amplitude.

Mean and standard deviation: The arithmetic mean and standard deviation (std) were calculated to represent the central tendency and dispersion of the signal amplitude, respectively. We also calculated the maximum and minimum values of the time domain EEG signal.

Skewness: This metric indicates the tendency for extreme values in one direction by measuring the asymmetry of the signal distribution around its mean.

(5) $$Skew\left(x_{i}\right)=\sqrt{\frac{\frac{1}{N}\left[\sum_{i=1}^{N}{\left(x_{i}-\mu \right)}^{3}\right]}{\left(\sigma^{3}\right)}}$$

Kurtosis: This is a statistical measure of an EEG signal’s complexity. Additionally, it describes whether the waveform is reasonably flat around its mean value or has a dramatic peak. Smaller values show a flatter profile close to the mean, while larger values show a more prominent peak at the mean.

(6) $$Kurtosis=\frac{E\left[{\left(x\left(n\right)-\mu \right)}^{4}\right]}{{\left[E\left[{\left(x\left(n\right)-\mu \right)}^{2}\right]\right]}^{2}}$$

where *µ* is the standard deviation, and *E* is the expected value estimator of the signal *x*(*n*).

#### 2.5.4. Hjorth Parameters

Hjorth parameters are time-domain features used for the EEG signal. These parameters, Activity, Mobility, and Complexity, offer low-computational-cost descriptors of signal variance and frequency [30,31].

Activity: Representing the signal power or variance of the time function y(t).

(7) $$Activity=\sum_{t=1}^{T}\frac{{\left(X\left(t\right)-\mu \right)}^{2}}{T}$$

Mobility: An estimate of the mean frequency, calculated as the square root of the ratio of the first derivative to the variance of the signal.

(8) $$Mobility=\sqrt{\frac{var\left(X^{\prime }\left(t\right)\right)}{var\left(X\left(t\right)\right)}}$$

Complexity: A measure of the deviation of the signal from a pure sine wave, defined as the ratio of the mobility of the first derivative to the mobility of the signal.

(9) $$Complexity=\frac{Mobility\left(X^{\prime }\left(t\right)\right)}{Mobility\left(X\left(t\right)\right)}$$

where *T* is the number of samples and *X*(*t*) is the EEG signal in the time domain. In this context, activity represents the overall power of the signal, mobility reflects its dominant or average frequency content, and complexity quantifies the degree of variation in frequency over time.

#### 2.5.5. Entropy Features

Nonlinear complexity measures were employed to quantify the randomness and information content of the EEG signals.

Shannon Entropy: This feature evaluates the uncertainty or impurity in the distribution of the signal values. For a probability distribution *p_i_* of signal amplitudes, it is defined as:

(10) $$H_{shannon}=-\sum p_{i}\log_{2}\left(p_{i}\right)$$

Spectral Entropy: This measure quantifies the complexity of the power spectrum. A flat spectrum (white noise) yields high entropy, while a peaked spectrum (pure tone) yields low entropy. It is calculated by normalizing the PSD to obtain a probability distribution and applying the Shannon entropy formula.

### 2.6. Feature Matrix Construction and Feature Selection

PSD metrics, band power, Hjorth parameters, temporal statistics, and entropy values were among the retrieved characteristics from the O1 and O2 channels that were combined into a single feature matrix, each row of which represented a subject’s entire EEG profile. The spectral, temporal, and nonlinear dynamics needed for downstream categorization were captured by this high-dimensional representation.

#### 2.6.1. Preprocessing and Normalization

To maintain numerical stability and prevent features with huge magnitudes (such as absolute power) from dominating the study, all variables were normalized with z-scores (StandardScaler). A zero mean and a unit variance were applied to each characteristic. This normalization was conducted within the cross-validation scheme’s training folds to prevent data leakage and ensure that all features contributed equally to distance-based and gradient-descent optimization techniques.

#### 2.6.2. Multicollinearity, Feature Selection, and Overfitting Mitigation

A multistage feature selection approach was used to minimize dimensionality and duplication while keeping the most discriminative variables due to the intrinsic connection between EEG measures.

Correlation Analysis: To measure monotonic connections, a Spearman rank correlation matrix was generated. To increase model stability, pairs of features showing severe collinearity (ρ > 0.9) were examined, and redundant variables were eliminated [32].

Hybrid Selection: Given the limited sample size relative to the extracted feature space, feature selection was used to reduce dimensionality and mitigate overfitting risk. We applied SelectKBest (ANOVA F-statistics) as a filter method to retain the most discriminative features and improve model stability. Higher-order statistics such as skewness and kurtosis were included to capture non-Gaussian signal characteristics and inter-subject variability, whereas more complex measures such as fractal dimensions or phase–amplitude coupling were not considered due to the limited sample size and the increased risk of overfitting associated with high-complexity features. Feature selection was performed using SelectKBest with ANOVA F-statistics, retaining the top k = 8 features within each training fold of the leave-one-subject-out (LOSO) validation framework [30]. This value was chosen as a balance between dimensionality reduction and information preservation in a small-sample setting. Random Forest feature importance was subsequently used to assess the relative contribution of the selected features and support interpretability, but not as a secondary elimination step.

### 2.7. Machine Learning Models

To classify participants into GD and HC groups based on the extracted occipital EEG features, several supervised learning algorithms were implemented. The classification framework was designed to handle the high dimensionality of the feature space and potential non-linear relationships that are inherent in EEG data.

#### 2.7.1. Models Used

**Figure 4:**
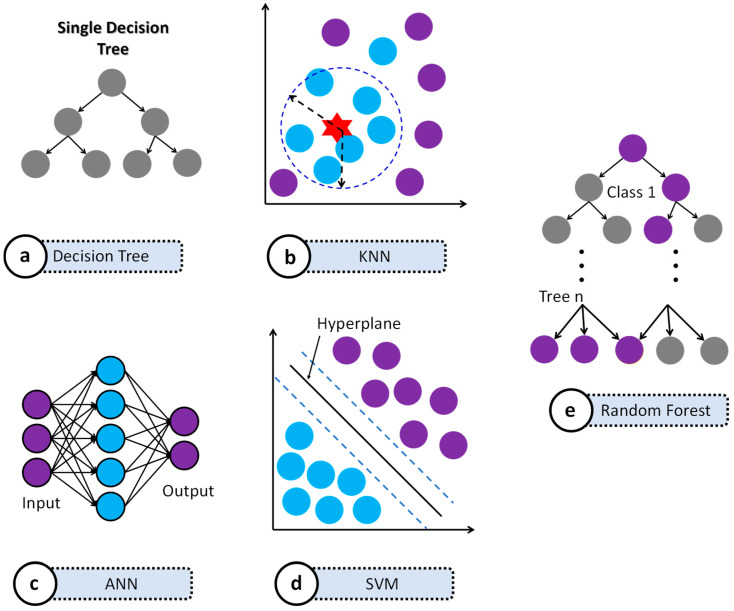
Visual representation of machine-learning classifiers used in this study. (**a**) Decision Tree: a hierarchical model that splits data based on feature thresholds. (**b**) K-Nearest Neighbors (KNN): classification of a query point (red star) based on its closest neighboring samples in the feature space. (**c**) Artificial Neural Network (ANN): a multilayer network that learns nonlinear relationships through interconnected neurons. (**d**) Support Vector Machine (SVM): a margin-based classifier that determines the optimal separating hyperplane using a kernel-transformed feature space. (**e**) Random Forest: an ensemble of multiple decision trees that vote to improve prediction robustness. Colored points represent samples from different classes (e.g., GD and HC) and are shown for conceptual illustration rather than quantitative results.

The following algorithms were selected based on their proven efficacy in prior EEG-based addiction and clinical classification studies and are illustrated in Figure 4.

Decision Tree: A decision tree recursively partitions the feature space into regions that are as homogeneous as possible with respect to the class label. At each internal node, a split is chosen that maximizes information gain or minimizes Gini impurity. For a split on feature x_j_ at threshold *t*, the Gini impurity is given as:

(11) $$G=\;\overset{K}{\underset{k=1}{\sum }}p_{k}\left(1-p_{k}\right)$$

where *p_k_* is the proportion of class *k* in the node. The model depicted in Figure 4a represents this hierarchical partitioning.

K-Nearest Neighbors (KNN): KNN is a non-parametric classifier that assigns a test sample to the majority class among its K nearest neighbors in feature space, as illustrated in Figure 4b. Distances were computed in the standardized feature space using the Euclidean metric. This model is suitable for low-dimensional EEG feature sets and can capture local structure in the data.

Artificial Neural Network (ANN): A Multilayer Perceptron (MLP) architecture was implemented to model complex nonlinear mappings between EEG features and GD status. The network consisted of an input layer, hidden layers with nonlinear activation functions (ReLU), and an output layer (Figure 4c).

For an input vector *x*, the network computes:

(12) $$h=\sigma \left(W_{1}x+b_{1}\right),\;\;\;\hat{y}=\sigma \left(W_{2}x+b_{2}\right)$$

where *σ* is a nonlinear activation function, **W_1_**, **W_2_** are weight matrices, and **b_1_**, **b_2_** are bias terms. Parameters are optimized by minimizing the cross-entropy loss with gradient-based optimization.

Support Vector Machine (SVM): This was included as a robust classifier for high-dimensional data. SVM constructs an optimal hyperplane (or set of hyperplanes) that maximizes the margin between the two classes, often using a kernel function (e.g., RBF) to handle non-linear separability (Figure 4d).

Random Forest: Random Forest extends the decision trees by training an ensemble of trees on bootstrap samples of the data, with random feature subsets considered at each split. The final prediction is obtained by majority vote across trees. RF has been frequently used in EEG-based behavioral disorder studies because it provides good accuracy and embedded feature importance while being robust to noise and correlated predictors (Figure 4e).

While CNN and LSTM networks achieve 90–98% accuracy in larger EEG studies, these deep learning approaches require substantially larger sample sizes to avoid overfitting [16,33]. Our proof-of-concept design (*N* = 30) necessitates simpler, interpretable classifiers. Decision Tree, KNN, and Random Forest provide transparent decision rules suitable for early screening tools.

#### 2.7.2. Hyperparameter Settings

Given the limited sample size and the use of a strict leave-one-subject-out (LOSO) validation framework, model hyperparameters were selected conservatively to limit model complexity and reduce overfitting rather than optimized through exhaustive grid search. Tree-based models were constrained in depth and splitting criteria to reduce variance, while k-nearest neighbor classification employed a fixed neighborhood size of five, as commonly adopted in EEG classification studies. The artificial neural network employed a single hidden layer comprising 16 units with a rectified linear activation function, selected to balance representational capacity and generalization in a small-sample context. For the support vector machine, a radial basis function kernel was used with a regularization parameter C = 1.0 and a standard kernel scaling. All hyperparameter values were kept constant across LOSO folds to ensure stability and reproducibility, and feature selection was performed exclusively within each training fold to prevent information leakage.

#### 2.7.3. Pipeline Construction

**Figure 5:**
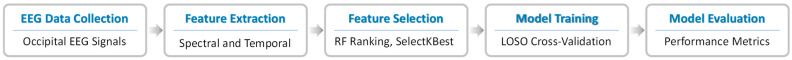
Overview of the machine learning pipeline used in this study, illustrating the sequential workflow from EEG data collection through feature extraction, feature selection, model training, and final model evaluation.

A systematic processing pipeline was constructed to prevent data leakage and ensure robust model training. A schematic overview of the complete machine learning pipeline is shown in Figure 5. To strictly prevent information leakage, all data transformation steps (StandardScaler, SelectKBest, recursive feature elimination (RFE)), implemented using scikit-learn (v1.7), were fitted only on the training set of the current LOSO iteration and then applied to the held-out test subject.

StandardScaler: Features were normalized to zero mean and unit variance to ensure scale invariance for distance-based models (KNN, SVM, MLP) [34].

Model Training: The subset of processed features was used to train the chosen ML classifier.

### 2.8. Model Evaluation

To ensure the generalizability and dependability of the results, the machine learning models’ performance was thoroughly assessed.

#### 2.8.1. Leave-One-Subject-Out (LOSO) Cross-Validation

Given the small sample size (*N* = 30), model evaluation was performed using a leave-one-subject-out (LOSO) cross-validation framework, in which all preprocessing, feature selection, and hyperparameter tuning were conducted exclusively within the training data of each fold. This method removes “data leakage” and gives a reasonable assessment of the model’s capacity to generalize to new, unobserved people [35].

Model performance was reported with uncertainty and subject-level robustness indicators rather than as a single point estimate. For each classifier, overall accuracy was computed as k/n, where n is the number of subjects and k is the number of correctly classified held-out subjects across LOSO folds. Uncertainty in accuracy was quantified using 95% confidence intervals based on the Wilson score method for binomial proportions. To capture variability across LOSO folds, we additionally report dispersion of per-subject LOSO outcomes (correct/incorrect) by analyzing the per-subject correctness outcomes (binary 0/1) across folds and computing the standard deviation (SD) and standard error of the mean (SEM). Finally, subject-level misclassification analysis was performed by listing, for each held-out subject, the true label and predicted label, allowing for the inspection of classifier failure modes (e.g., GD → HC vs. HC → GD errors). For LOSO with *N* = 30 subjects, this resulted in 30 binary per-subject predictions (correct/incorrect across the 30 held-out test folds), from which we computed overall accuracy, fold-wise SD, and Wilson CI.

To assess whether classification performance exceeded chance level under small-sample conditions, permutation testing was performed within the LOSO cross-validation framework. Class labels were randomly permuted across subjects while preserving the LOSO structure, and classification accuracy was recomputed for each permutation. This procedure was repeated 5000 times to generate a null distribution of accuracies. The permutation *p*-value was calculated as P[acc_perm ≥ acc_obs]. This non-parametric approach does not rely on distributional assumptions and is well-suited for small-sample EEG studies [36,37].

#### 2.8.2. Performance Metrics

Model performance was quantified using accuracy, precision, recall, and F1-score. Moreover, model efficacy was further assessed using standard binary classification metrics derived from the number of True Negatives (TN), True Positives (TP), False Negatives (FN), and False Positives (FP). The matrices are defined as:

Accuracy: The total proportion of cases that were correctly classified.

(13) $$\mathrm{Accuracy}=\frac{\left(\mathrm{TP}+\mathrm{TN}\right)}{\left(\mathrm{TP}+\mathrm{TN}+\mathrm{FP}+\mathrm{FN}\right)}$$

Precision: The proportion of true positive predictions among all positive predictions.

(14) $$\mathrm{Precision}=\frac{\mathrm{TP}}{\mathrm{TP}+\mathrm{FP}}$$

Recall (Sensitivity): The ability of the model to identify all actual positive cases.

(15) $$\mathrm{Precision}=\frac{\mathrm{TP}}{\mathrm{TP}+\mathrm{FN}}$$

F1-Score: The harmonic means of precision and recall, providing a balanced metric for uneven class distributions.

(16) $$F1-\mathrm{Score}=2\times \frac{\mathrm{Precision}\times \mathrm{Recall}\;}{\mathrm{Precision}+\mathrm{Recall}}$$

#### 2.8.3. Confusion Matrices

Confusion matrices were calculated for each classifier by combining predictions from all test folds. The confusion matrix summarizes the counts of true positives, true negatives, false positives, and false negatives, as well as allowing for visual evaluation of class-specific performance.

### 2.9. Software & Tools

Python 3.7 was used to develop the complete computational framework, taking advantage of its vast ecosystem for machine learning and scientific computing. EEGLAB (v2025.1), a popular open-source toolkit for electrophysiological signal processing, was utilized in the MATLAB (Version R2024b, MathWorks, Natick, MA, USA) environment to perform data preparation, including artifact removal and filtering. Custom Python scripts that used Pandas for data structuring and NumPy for numerical operations were used to extract features and create matrices. Scikit-learn (v1.7) was used to construct the machine learning pipeline, which included feature selection, model training, and performance evaluation. Along with tools for cross-validation (StratifiedKFold), data standardization (StandardScaler), and metric computation (accuracy, F1-score), this library offered reliable implementations of the classifiers (SVM, Random Forest, KNN, MLP).

## 3. Results

### 3.1. Participant Characteristics

A total of 30 participants were included in the study, divided into two equal groups: 15 individuals classified as GD mobile video gamers and 15 HCs. All participants were university students and adults; no minors, neurological patients, or individuals with a history of psychiatric illness were included.

**Figure 6:**
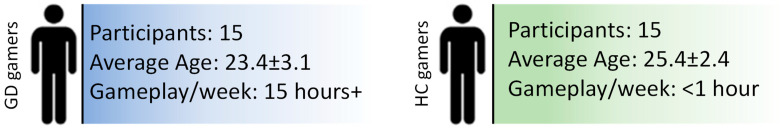
Demographic characteristics of the study participants.

The GD group had a mean age of 23.4 ± 3.1 years, whereas the HC group had a mean age of 25.4 ± 2.4 years. Gender distribution across the full dataset consisted of 43.3% females and 56.7% males, with no significant difference between groups. The demographic distribution for both groups is summarized visually in Figure 6.

### 3.2. EEG Signal Integrity Assessment

#### 3.2.1. Raw EEG Visualization

**Figure 7:**
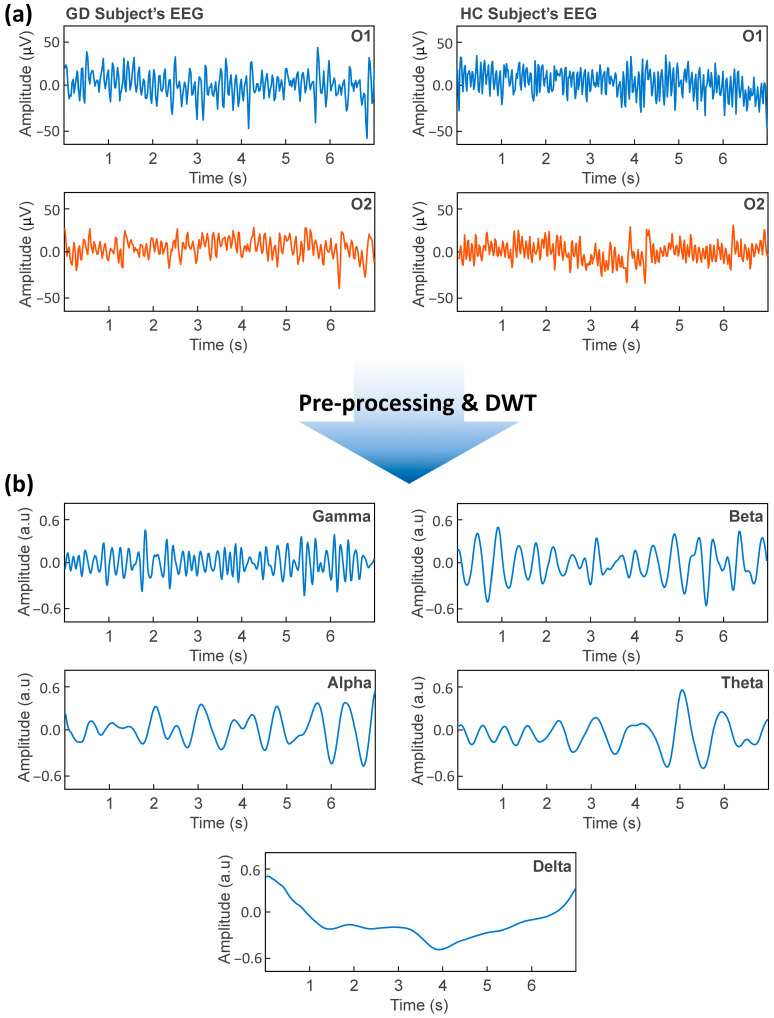
Representative EEG signals from occipital electrodes during mobile gameplay. (**a**) The top left panel shows raw O1 and O2 EEG signals from a participant with Gaming Disorder (GD), and the top right panel shows corresponding EEG signals from a Healthy Control (HC). Both groups exhibit physiologically plausible occipital EEG activity during task engagement, without major artifacts. Following preprocessing, the signals were decomposed using the Daubechies-4 Discrete Wavelet Transform. (**b**) The lower panels illustrate the reconstructed Gamma, Beta, Alpha, Theta, and Delta components, exhibiting successful isolation of frequency-specific rhythms for subsequent feature extraction.

All EEG recordings were collected while participants played a mobile video game, which required continuous visual attention and sensorimotor engagement. The Raw O1 and O2 traces were visually inspected to ensure adequate physiological quality before feature extraction. As shown in Figure 7a, both GD and HC participants showed typical occipital EEG patterns associated with visually demanding tasks. The raw O1 and O2 traces showed typical occipital alpha-dominant morphology. The remaining segments had steady voltage fluctuations following artifact rejection, demonstrating that, despite the mobility of the task, the dry electrode contacts maintained adequate impedance stability for spectral analysis.

Amplitudes were confirmed to remain within acceptable EEG signal bounds due to natural head and ocular movements during games. Artifacts were removed via manual noise removal.

#### 3.2.2. Artifact Rejection Summary and Data Retention

After excluding the initial and final 20 s of each recording, a fixed duration of 560 s of EEG data per participant was available for analysis. Artifact rejection was performed using a predefined peak-to-peak amplitude threshold of ±100 µV, applied uniformly across all subjects. Under this criterion, the mean rejected duration was 63.14 ± 9.25 s in the gaming disorder (GD) group and 59.27 ± 14.21 s in healthy controls (HC), corresponding to 11.28 ± 1.65% and 10.58 ± 2.54% of the analyzed data, respectively (Table 1). The retained signal duration, therefore, exceeded 88% in both groups (GD: 496.86 ± 9.25 s, HC: 500.73 ± 14.21 s), ensuring sufficient data length for stable spectral estimation, including low-frequency components. No significant group difference in rejected duration was observed at this threshold (Welch’s t(24.1) = 0.88, *p* = 0.385), indicating comparable artifact burden between groups.

To further assess robustness and address potential subjectivity in amplitude-based artifact handling, a sensitivity analysis was conducted using stricter (±75 µV) and more permissive (±125 µV) thresholds (Table 2). As expected, stricter thresholds resulted in higher rejection rates, while more permissive thresholds retained a greater proportion of data in both groups. Across all thresholds, rejection percentages remained within a moderate range (approximately 6–19%), and no statistically significant group differences in rejected duration were detected (all *p* > 0.05). Importantly, the relative balance between retained and rejected data was preserved across thresholds, demonstrating that preprocessing did not introduce systematic group-dependent bias.

#### 3.2.3. Validation of Wavelet-Based Frequency Decomposition

The EEG signals were decomposed into constituent frequency bands using the Daubechies-4 Discrete Wavelet Transform to extract task-related oscillatory components. Figure 7b shows the reconstructed Gamma, Beta, Alpha, Theta, and Delta signals for an example subject. Fast and medium-frequency oscillations linked to visual processing, cognitive load, and motor engagement were observed in the gamma and beta bands. Wavelet decomposition verified robust separation of EEG frequency bands, supporting the reliability of subsequent band-specific spectral and machine-learning analyses. These findings validate the use of the decomposition pipeline for downstream feature extraction, such as band power, temporal descriptors, entropy measures, and PSD-based statistics, by confirming that it successfully extracted significant frequency-specific brain activity during interactive gameplay.

### 3.3. PSD (Power Spectral Density) Analysis of Occipital Rhythms

**Figure 8:**
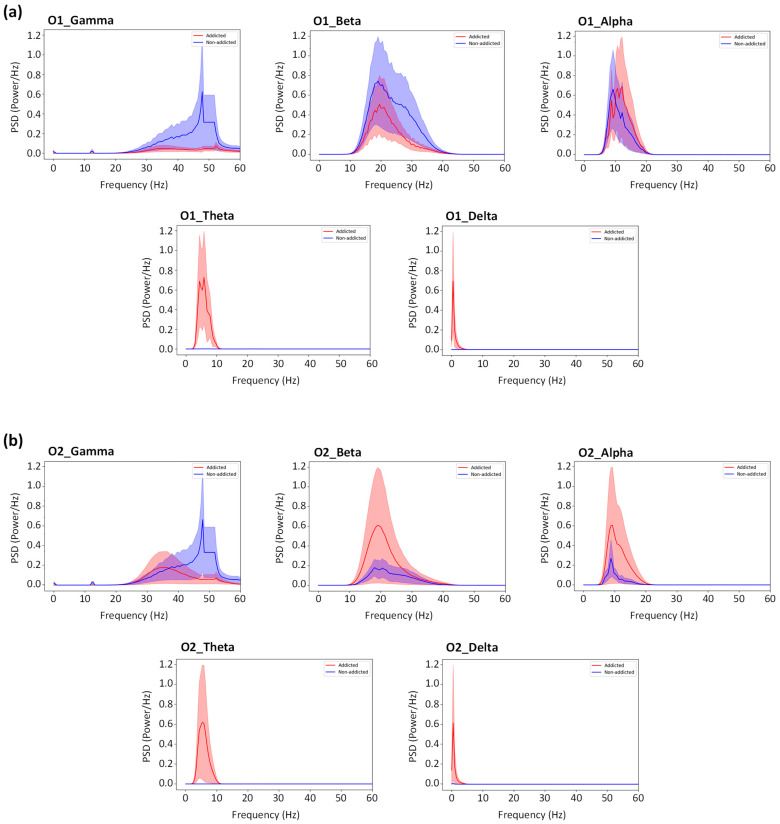
Power spectral density (PSD) distributions for GD and HC groups across O1 and O2 channels. Mean power spectral density (PSD) of occipital channels (**a**) O1 and (**b**) O2 for GD (red; *n* = 15) and HC (blue; *n* = 15) participants, recorded during mobile gameplay. Each sub-panel represents one canonical EEG frequency band—gamma (30–45 Hz), beta (13–30 Hz), alpha (8–13 Hz), theta (4–8 Hz), and delta (1–4 Hz)—with solid lines indicating group means and shaded regions showing ± standard error. Across both O1 and O2, GD gamers exhibit markedly elevated delta, theta, and alpha power compared with HC gamers, whereas HC participants display relatively stronger beta and broadband gamma activity. Together, these spectra indicate a bilateral shift toward slow-frequency dominance over the occipital cortex in the GD group, aligning with previously reported quantitative EEG abnormalities in gaming disorder.

Figure 8 shows clear spectral power differences between the GD and HC groups in the occipital visual regions (O1 and O2) based on the analysis of EEG signals recorded during active video gaming sessions. Following preprocessing, PSDs were extracted and summarized for the delta, theta, alpha, beta, and gamma bands. Group means are shown by solid lines, while between-subject variability is shown by shaded areas. While HC players displayed comparatively higher fast-frequency activity, GD participants displayed a typical shift toward increased slow-frequency activity across both channels. The electrophysiological changes associated with Internet gaming disorder are consistent with this trend.

#### 3.3.1. Delta and Theta Bands

The delta (1–4 Hz) and theta (4–8 Hz) ranges showed the greatest group differences. Slow-wave power was significantly higher in GD participants at both O1 and O2 (Welch’s two-sample *t*-test, *p* < 0.05), while HC participants exhibited lower delta and theta power. This increased slow-frequency activity has been associated with decreased cortical arousal and impaired top-down control, and it is consistent with previous quantitative electroencephalography (QEEG) studies showing greater delta/theta power in individuals with gaming-related compulsive behaviors. QEEG has been widely applied to characterize neural oscillatory patterns associated with behavioral addictions. Importantly, these effects appeared during active gameplay, suggesting that slow-wave dominance is not exclusive to resting-state circumstances.

#### 3.3.2. Alpha Band

In the alpha range (8–13 Hz), GD participants again showed consistently higher power across both occipital sites. Elevated alpha levels during a visually demanding activity could indicate lower flexibility in visual attention systems or more habitual/automatic processing of game inputs. Previous task-based EEG research in gaming disorder groups has found aberrant alpha modulation, particularly in the parieto-occipital region.

#### 3.3.3. Beta Band

Beta activity (13–30 Hz) demonstrated more moderate and regionally varied patterns. HC participants had higher beta power at O1, although differences were smaller at O2. Stronger beta power in HC gamers may suggest more effective visual–attentional processing because beta oscillations are linked to prolonged attention and sensorimotor engagement. Beta differences may be secondary features because these impacts were not as strong as those seen in the slow bands.

#### 3.3.4. Gamma Band and Line Noise

In the gamma range, both channels showed a narrow peak at 50 Hz, reflecting residual line noise rather than neural activity. Accordingly, gamma-band analyses were restricted to 30–45 Hz, fully excluding the line-noise region. Within this 30–45 Hz range, gamma power was relatively low with only modest group differences, and all interpretations of gamma activity were confined to this line-noise–free band.

Contrary to the heightened high-frequency arousal typically expected during intense visuomotor tasks, the GD group exhibited a distinct pattern of spectral slowing. Specifically, the GD group showed significantly lower Beta band (15–30 Hz) power compared to the HC group (Figure 8a). While HC gamers maintained high Beta activity consistent with sustained visual attention, the suppression of Beta power in the GD group suggests diminished top-down cortical activation. This aligns with previous findings linking reduced Beta power to deficits in inhibitory control and attentional stability in Internet Gaming Disorder [23,38].

Simultaneously, the GD group showed considerably higher power in the slow-frequency Delta (0.5–4 Hz) and Theta (4–8 Hz) bands throughout both O1 and O2. Instead of cognitive attention, increased theta power during gaming has been connected to processing related to cravings and emotional dysregulation [39]. High Delta and Theta Power suggest that the GD brain may operate in a “neural efficiency” mode, where habitual task performance requires less cortical resource recruitment than in non-addicted controls. Alternatively, this slow-wave dominance could reflect sensory gating, where the brain filters out non-game stimuli to maintain a trance-like focus [38].

Additionally, the GD group exhibited elevated Gamma band (30–45 Hz) power (Figure 8). While high-frequency Gamma is typically associated with active binding of visual information, its elevation in the context of reduced Beta (hypoarousal) has been identified as a specific marker of dysfunctional reward sensitivity and sensory craving in GD [9,40]. This suggests that while the GD brain is cognitively “under-engaged” (low Beta), it is simultaneously “hyper-reactive” to the reward/sensory aspects of the game (high Gamma).

Finally, the GD group displayed a significantly higher Alpha band (8–13 Hz) peak (Figure 8). High Alpha power during a visual task is atypical for healthy attention (which usually involves alpha desynchronization) and suggests that GD gamers perform the task in a highly automatized, dissociated state requiring less active cortical processing resources compared to the HC group [38,41].

### 3.4. Temporal and Nonlinear EEG Feature Analysis

**Figure 9:**
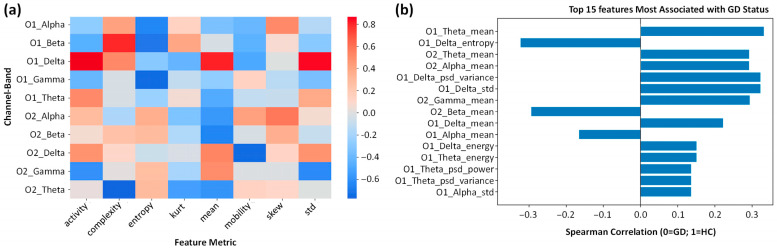
Temporal and Nonlinear Feature Analysis. (**a**) Heatmap of Cohen’s d effect sizes comparing GD vs. HC groups across Hjorth parameters and statistical moments. Warm colors (red) indicate higher values in the GD group, while cool colors (blue) indicate lower values. Notably, the GD group exhibits increased Delta activity/variance (red) but reduced complexity in Alpha/Theta bands (blue), reflecting a high amplitude yet simplified neural state. (**b**) Top 15 EEG features most strongly associated with disordered behavior status (Spearman’s correlation). Bars pointing right (positive) indicate features that are elevated in the GD group (e.g., Theta Mean, Delta Variance), while bars pointing left (negative) indicate features that are suppressed (e.g., Beta Mean, Delta Entropy). This profile highlights a shift toward high-amplitude slow waves with reduced signal complexity and diminished arousal (Beta) in GD gamers.

To capture temporal characteristics of the band-limited EEG signals, we computed classical summary statistics—mean, standard deviation, skewness, and kurtosis—as well as nonlinear descriptors including Hjorth parameters (activity, mobility, complexity) and entropy-based measures (Shannon and spectral entropy). Group differences between GD and HC subjects were quantified using Cohen’s d, and results are visualized in Figure 9a. Spearman correlation analysis revealed the specific features most discriminative of GD status (Figure 9b).

Consistent with the spectral slowing observed in the PSD analysis, features related to slow-wave activity showed the strongest associations. O1 Delta Variance and O1 Delta Standard Deviation exhibited strong positive correlations with the GD label. This indicates that the EEG signals of GD gamers are characterized not only by higher Delta amplitude but also by significantly greater temporal variability in these slow rhythms. This instability in slow-wave oscillations may reflect the fluctuating cortical arousal levels associated with the “zoned-out” or trance-like state of GD.

In the nonlinear domain, O1 Delta Entropy showed a significant association. The reduced entropy in the GD group suggests a loss of signal complexity, pointing to a more rigid, deterministic pattern of neural firing. This “regularization” of brain activity (high amplitude, low complexity) is often a hallmark of pathological states where the rich, chaotic dynamics of healthy cognition are replaced by hypersynchronous idling rhythms.

Additionally, Hjorth Complexity parameters in the Theta and Alpha bands were lower in the GD group (Figure 9a), further corroborating the loss of neural complexity. The combination of high slow-wave variance and low signal complexity effectively fingerprints the GD brain state as one of high-energy but low-information processing.

### 3.5. Statistical Feature Comparison and Effect Size Analysis

To quantify group differences across the extracted EEG features, we performed independent *t*-tests and calculated Cohen’s d effect sizes. Given the exploratory nature of the high-dimensional feature set, false discovery rate (FDR) correction was applied. While no single feature reached statistical significance after FDR adjustment (p_FDR > 0.05), likely due to the sample size, several features exhibited large effect sizes (|d| > 0.8) and near-threshold uncorrected *p*-values (*p* < 0.10), indicating strong practical significance despite the lack of statistical significance after multiple comparison correction (Table 3).

The strongest group differences were observed in the Delta band (0.5–4 Hz) dynamics. The GD group showed substantially higher O1_Delta_Activity (d = 0.88, *p* = 0.067) and O1_Delta_Standard_Deviation (d = 0.84, *p* = 0.073) compared to the HC group. These large effect sizes suggest that high-amplitude, variable slow-wave activity is a robust characteristic of the GD brain state, reinforcing the spectral slowing patterns observed in the PSD analysis.

Additionally, trends were observed in nonlinear complexity measures. O1_Beta_Complexity showed a large positive effect size (d = 0.80), while O2_Theta_Complexity (d = −0.77) and O1_Gamma_Entropy (d = −0.75) showed medium-to-large negative effect sizes. This divergence implies that while high-frequency activity (Beta) may become more complex/irregular in GD gamers, the lower frequency rhythms (Theta) and broadband Gamma activity become more predictable or regularized.

Although these findings require validation in larger cohorts, the magnitude of the effect sizes (d > 0.8) highlights Occipital Delta Activity and Signal Variability as the most promising candidate biomarkers for distinguishing gaming disorder. In small sample biomedical studies, large effect sizes (d > 0.8) are considered clinically meaningful and often more informative than *p*-values alone [42,43].

To assess whether concatenation of artifact-free segments influenced feature estimation, group comparisons were repeated using a non-concatenated, segment-wise feature extraction pipeline as a validation analysis (Table 4). Across key low-frequency PSD-derived features, including delta activity and delta variability, group-level trends and effect size magnitudes were preserved relative to the concatenated analysis. Cohen’s d values remained large (|d| ≈ 0.9), and *p*-values showed comparable magnitudes. Although group-level trends and large effect sizes for low-frequency PSD-derived features were preserved across aggregation strategies (e.g., delta activity: Cohen’s d = 0.88 with concatenation vs. d = 0.90 without concatenation), some higher-frequency and complexity-related features exhibited modest quantitative variation. Low-frequency PSD estimates are less sensitive to boundary discontinuities because they integrate power over longer time scales relative to the segment length. Notably, gamma-band entropy showed reduced effect size and loss of statistical significance without concatenation (d = −0.75 vs. −0.51, *p* = 0.124), demonstrating that concatenation can alter the estimation of certain higher-frequency and complexity-related features. However, the robustness of low-frequency features (our primary findings) to concatenation choice indicates these main results are not attributable to concatenation artifacts.

### 3.6. Feature Importance and Correlation Analysis

**Figure 10:**
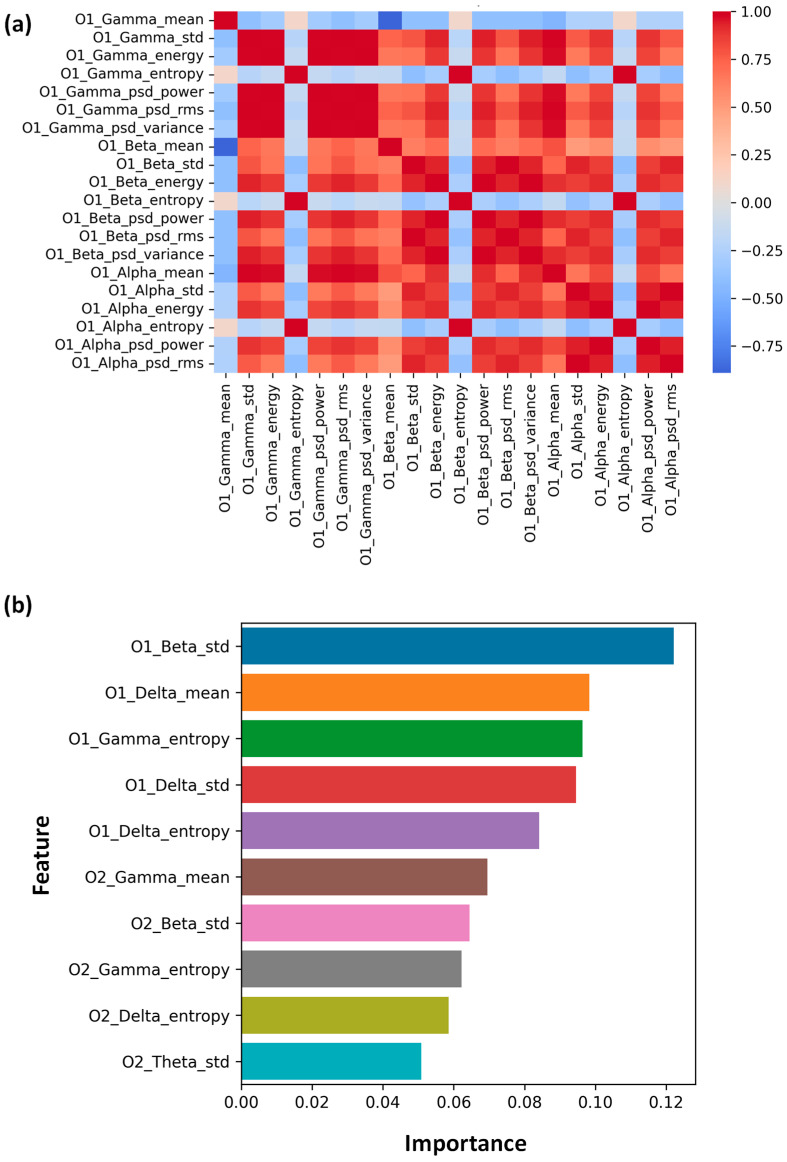
Feature Importance and Correlation Analysis. (**a**) The extracted EEG features’ Spearman correlation heatmap. Blue blocks show negative relationships, whereas red blocks show significant positive correlations, suggesting redundant feature groups (such as within-band power measures). (**b**) Top 10 discriminative features ranked by Random Forest Gini importance. The single most significant predictor, O1_Beta_std (beta variability), is followed by O1_Delta_mean and O1_Gamma_entropy, underscoring the critical role that slow-wave amplitude and beta instability play in categorizing gaming addiction.

To identify the most critical EEG markers for classifying GD and to evaluate inter-feature relationships, we performed a feature importance analysis using a Random Forest classifier, complemented by a Spearman correlation heatmap to assess multicollinearity (Figure 10).

#### 3.6.1. Feature Correlation Analysis

The Spearman correlation heatmap (Figure 10a) revealed distinct clusters of covarying features, which informed our interpretation of the feature space.

Strong Intra-Band Correlations: Features generated from the same frequency band, such as Gamma_mean, Gamma_energy, and Gamma_psd_power, demonstrated nearly perfect positive correlations (r > 0.9, red blocks), as expected. This validates our use of feature selection (SelectKBest) to reduce dimensionality by confirming that these metrics capture redundant information.

Cross-Band Independence: Importantly, the top feature, Beta_std, showed relatively modest correlations with the Delta and Alpha features. This suggests that the Random Forest model emphasized beta variability because it provides distinct information not captured by the dominant slow-frequency fluctuations.

Inverse Relationships: Negative correlations (blue) were observed between some power and complexity metrics, supporting the view that high-amplitude rhythmic states (high power) often correspond to lower signal complexity (low entropy).

#### 3.6.2. Random Forest Feature Importance

The Random Forest model identified O1_Beta_Std (standard deviation of Beta power) as the most discriminative feature (importance ~ 0.12), followed by O1_Delta_mean and O1_Gamma_entropy (Figure 10b).

The emergence of Beta variability as the top predictor is highly significant. While previous sections discussed decreased mean Beta power (hypoarousal), this research implies that the instability or variability of Beta activity is an even stronger indicator of the GD gamer’s brain activity. The high fluctuation in Beta rhythms may represent unstable cognitive control and variable attention levels, corresponding to the “disengaged” state of Gaming Disorder (GD) [38]. Our spectral analysis is consistent with the high ranking of Delta mean power, indicating that increased slow-wave activity is a reliable, key feature of GD. The significance of Gamma entropy supports the “high energy, low information” theory by indicating that GD gamers have a different level of high-frequency processing complexity, which is associated with impulsivity and reward-seeking behavior.

### 3.7. Machine Learning Performance

**Figure 11:**
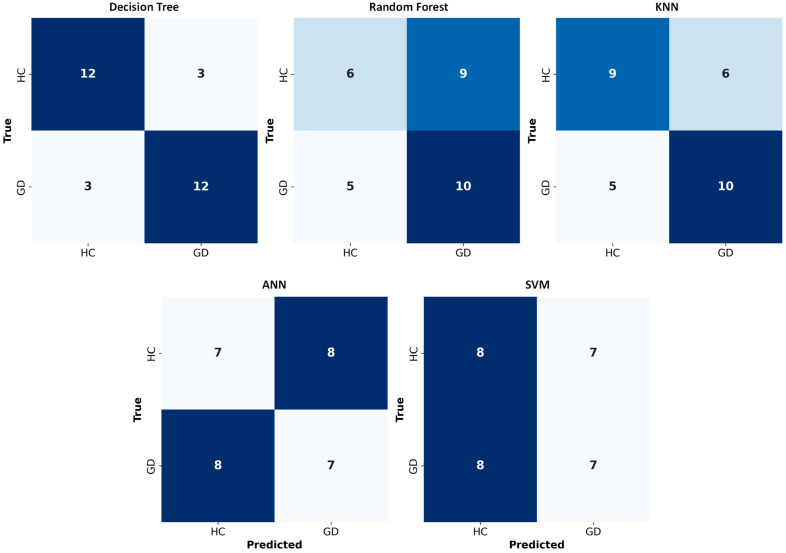
Aggregated Confusion Matrices for Leave-One-Subject-Out (LOSO) Validation. The matrices display the cumulative prediction results for all 30 participants (15 Healthy Controls, 15 Gaming Disorder) across five machine learning models: (Top Left) Decision Tree, (Top Middle) Random Forest, (Top Right) K-Nearest Neighbors, (Bottom Left) Artificial Neural Network, and (Bottom Right) Support Vector Machine. The Decision Tree classifier achieved the highest accuracy (80.0%), correctly identifying 24 out of 30 subjects (12 HC, 12 GD), demonstrating superior sensitivity and specificity compared to the other models that struggled to generalize beyond random chance (Accuracy ≈ 50–63%). Color intensity represents the number of samples in each cell, with darker shades indicating higher counts.

A Leave-One-Subject-Out (LOSO) cross-validation strategy was used to assess the generalization capability of five machine learning models. The quantitative results (Figure 11 and Table 5) demonstrated that the interpretable Decision Tree model achieved the highest subject-level accuracy (24/30, 80.0%) with the narrowest 95% confidence interval, outperforming both distance-based (KNN) and complex ensemble and deep learning architectures.

#### 3.7.1. Model Evaluation and Comparison

The Decision Tree proved to be the best-performing model in this small-sample study, achieving the highest subject-level accuracy (24/30, 80.0%) with the lowest standard error (SEM = 0.075) among all classifiers (Table 5). This finding suggests that, in this dataset, a small set of threshold-like decision rules captured discriminative differences between GD and HC participants.

The K-Nearest Neighbors classifier outperformed random chance with a moderate accuracy of 19/30 subjects (63.3%), as reported in Table 5, but it was unable to match the Decision Tree’s robustness. This suggests that although there is some local clustering, specific rules are a better way to capture the decision boundary than proximity.

Random Forest, SVM, and ANN performed near chance level, with accuracies ranging from 14/30 to 16/30 subjects (46.7–53.3%), accompanied by wide 95% confidence intervals and higher standard errors (Table 5). The large gap between their theoretical capacity and actual performance suggests limited generalization, potentially due to overfitting and/or model instability, a known limitation when applying high-variance models to datasets with high dimensionality relative to sample size (*N* = 30).

The Support Vector Machine (SVM) using the RBF kernel was unable to discriminate between groups, with an accuracy of 15/30 subjects (50.0%) and an F1-score of 0.483 (Table 5). This near-chance performance highlights the model’s vulnerability to the “curse of dimensionality” in small-N regimes, implying that the intricate, non-linear hyperplane needed to divide the classes in high-dimensional space could not be reliably inferred from the sparse training data.

The Artificial Neural Network performed marginally worse than chance due to its poor generalization (Accuracy = 14/30 subjects (46.7%), F1 = 0.467; Table 5). This demonstrates that the deep learning architecture lacked enough examples to converge on a meaningful solution, leading to model instability and overfitting.

#### 3.7.2. Performance Metrics Analysis

Table 5 quantitatively summarizes model trade-offs in terms of subject-level accuracy (k/*n*), dispersion (SEM), and uncertainty (95% Wilson confidence intervals). Results demonstrate that the Decision Tree successfully minimized both false positives and false negatives, unlike KNN (Accuracy 63.3%), which exhibited moderate performance but failed to capture the decision boundary as effectively.

The success of the Decision Tree suggests that the neurophysiological signatures of GD (e.g., elevated Delta power and Beta instability) are best characterized by hierarchical, rule-based thresholds rather than distance-based clusters. This finding is particularly relevant for clinical translation, as decision trees provide transparent, interpretable “if-then” rules (e.g., ‘If Delta > Threshold, then GD’) that align with the identified biomarkers [44].

#### 3.7.3. Stability and Reliability Analysis

**Figure 12:**
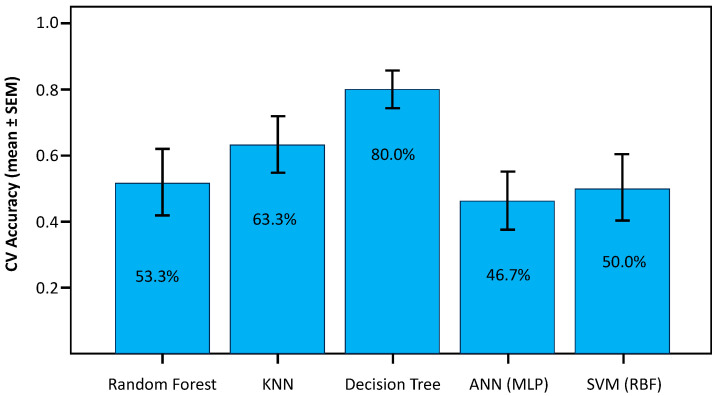
Cross-Validation Performance Comparison (*N* = 30). Bar chart displaying the classification accuracy (±standard error) of five machine learning models validated using the Leave-One-Subject-Out (LOSO). The Decision Tree classifier achieved the highest accuracy (24/30, 80.0%) with the lowest variability (SEM = 0.075). Error bars represent the SEM of subject-level correctness across LOSO folds, indicating relative stability of model performance across held-out subjects.

Figure 12 summarizes classification accuracy together with the SEM of subject-level correctness to illustrate variability in performance across held-out subjects under LOSO validation. Lower SEM reflects reduced variability in subject-level predictions, whereas higher SEM indicates greater dispersion of correct and incorrect classifications across individuals. The Decision Tree exhibited the lowest variability (SEM = 0.075), followed by KNN (SEM = 0.088), while Random Forest, SVM, and ANN showed higher variability (SEM ≈ 0.091).

Permutation testing (5000 permutations) further demonstrated that the Decision Tree classifier achieved performance significantly above chance (*p* = 0.0025). Although KNN also reached statistical significance (*p* = 0.0099), its lower subject-level accuracy (19/30, 63.3%) and higher misclassification rate compared with the Decision Tree indicate reduced practical robustness. Accordingly, subsequent analyses focused on the Decision Tree model, which combined higher accuracy with more balanced error patterns. In contrast, Random Forest (*p* = 0.0909), SVM (*p* = 0.3520), and ANN (*p* = 0.6436) did not achieve statistical significance, indicating chance-level performance in this small-sample setting.

In this specific context, our feature selection process narrowed the input down to just a few powerful biomarkers (especially Delta Power and Beta Variability) and created a scenario where a simple, decisive threshold was sufficient for classification. This aligns with findings where simpler models have been shown to outperform more complex ones on small sample biomedical data, especially after aggressive feature selection [45]. The failure of the complex models (ANN, SVM) to generalize is likely a classic case of overfitting, a known risk when applying high-capacity classifiers to datasets with a small sample-to-feature ratio.

## 4. Discussion

The current study used task-based EEG signals recorded from the occipital area to identify specific neurophysiological biomarkers of Gaming Disorder (GD). We identified a distinct neural signature in GD gamers that is marked by altered spectral dynamics, attentional variability, and potential neural habituation. By integrating spectral power analysis, nonlinear complexity measurements, and machine learning classification, we demonstrated that task-based occipital EEG can effectively distinguish GD subjects. Given the pilot study cohort, the achieved classification accuracy should be interpreted as a proof-of-concept demonstrating the feasibility of task-based occipital EEG for gaming disorder assessment, rather than as a definitive diagnostic benchmark.

### 4.1. Spectral Slowing: Neural Efficiency or Sensory Gating?

A primary finding was the significant elevation of Delta (0.5–4 Hz) and Theta (4–8 Hz) power in the GD group, accompanied by a suppression of Beta (13–30 Hz) activity. While often interpreted as “hypoarousal” in resting-state studies, in the context of active gameplay, this profile may reflect “Neural Efficiency” or “Automaticity”. In comparison to controls, experienced gamers (GD group) may interpret the complex visual inputs with less cerebral effort, thereby “automating” the gameplay. Alternatively, the high slow-wave power may be a sign of “sensory gating,” a condition sometimes referred to as “a trance or flow state” in which the brain suppresses processing of non-gaming ambient cues to retain focus. In contrast, the control group’s strong beta power indicates that they were actively and diligently focusing on the new assignment [46].

### 4.2. Beta Variability and Attentional Instability

Beyond mean power changes, our feature importance analysis identified O1 Beta Variability (Standard Deviation) as the single most discriminative feature. This extends current understanding by suggesting that GD pathology involves not just low attention, but unstable attention. High variability in Beta rhythms reflects fluctuations in the neural networks controlling visual vigilance. For the GD gamers, this implies a struggle to maintain consistent alertness, vacillating between focused attention and distractibility. This neural instability likely underpins the “attentional fluctuation” phenotype, where addicts struggle to maintain consistent top-down control even during preferred activities [41].

### 4.3. Alpha Synchronization and Sensory Gating

In occipital channels, the GD group exhibited a stronger alpha-band peak (8–13 Hz). While normally associated with inhibition, in this high-intensity context, it most likely represents “sensory gating”—a mechanism in which the trained brain actively blocks processing of task-irrelevant peripheral inputs to maintain attention. On the other hand, it might point to a condition known as “High-Alpha Flow,” in which prolonged gaming expertise enables GD participants to process game graphics with less cortical activation than controls [41].

### 4.4. Machine Learning Implications

To improve interpretability, we therefore examined dispersion of per-subject LOSO outcomes (correct/incorrect) and subject-level misclassification patterns (Table 6). Uncertainty/variability of subject-level correctness is summarized by SEM in Table 5/Figure 12, while Table 6 details error directions. This analysis shows that models with similar mean accuracy can behave differently at the subject level—for example, differing in the proportion of participants misclassified and in the direction of errors (GD → HC versus HC → GD). Directional errors directly map to class-conditional performance (GD → HC reduces GD sensitivity; HC → GD reduces HC specificity). Such error asymmetries are important because GD → HC errors correspond to missed detections, whereas HC → GD errors correspond to false alarms, which may have different implications depending on the intended screening or monitoring use case.

In the present study, the Decision Tree model exhibited the lowest proportion of misclassified subjects (20.0%) and achieved statistically significant performance under permutation testing (*p* = 0.0025), indicating the highest subject-level robustness among the evaluated classifiers. Importantly, its misclassification errors were not strongly skewed toward a single error direction (4 GD → HC vs. 2 HC → GD), suggesting no extreme directional bias in this dataset. In contrast, the Random Forest, KNN, ANN, and SVM models misclassified between 36.7% and 53.3% of subjects, reflecting substantially reduced robustness under LOSO validation. These models also showed a higher absolute number of GD → HC errors, indicating an increased tendency toward missed detections of GD cases, which is undesirable in screening-oriented applications. If the model is intended for screening, GD → HC errors are more costly; for confirmatory diagnosis, both error types matter. Overall, although several classifiers achieved moderate mean accuracy, the subject-level failure-mode analysis demonstrates that the Decision Tree model provides the most reliable and balanced performance across individuals in this small-sample setting.

In contrast to recent CNN-based EEG studies that emphasize end-to-end representation learning from large datasets, the present findings suggest that, in small-sample task-based EEG, low-frequency spectral features combined with rule-based classifiers may provide more stable subject-level generalization. This aligns with prior work showing that deep models excel when spatial richness and data volume are sufficient, whereas simpler models offer robustness and interpretability under constrained conditions [14,16].

The effectiveness of a single tree suggests that straightforward, hierarchical rules (such as specific cut-offs for Delta power and Beta variability) rather than intricate proximity clusters or high-dimensional hyperplanes are the best way to distinguish between GD and HC participants. This supports recent claims that on small, high-dimensional biomedical datasets, simpler, non-parametric models frequently perform better than deep learning [47].

### 4.5. Task-Based Occipital EEG in Relation to Prior Resting-State and Frontal Findings

Prior resting-state EEG and neuroimaging studies of gaming disorder have consistently reported spectral slowing, characterized by increased delta and theta activity and reduced higher-frequency power, across distributed cortical regions [23,38,48]. The present task-based occipital EEG findings suggest that similar spectral patterns are also expressed dynamically during active gameplay, indicating that such alterations are not limited to resting-state conditions but may emerge during sustained visual engagement and reward processing.

Whereas frontal EEG studies have primarily emphasized impairments in executive control and inhibitory regulation, the current occipital-focused approach highlights complementary disruptions in visual–attentional processing [41]. This regional complementarity supports models of gaming disorder as a distributed network dysfunction involving both executive and sensory-perceptual systems, rather than a deficit localized to a single cortical region.

Importantly, the proposed association between increased low-frequency occipital activity and neural efficiency or automatized visual processing should be interpreted as a hypothesis, therefore requiring further validation in larger cohorts and through complementary approaches such as high-density EEG, multimodal neuroimaging, or task manipulations explicitly probing executive control.

From a bioengineering perspective, this study demonstrates the feasibility of combining low-cost, wireless EEG hardware with a carefully designed signal processing and machine-learning framework for behavioral addiction assessment. By using dry electrodes and a minimal channel configuration during a naturalistic mobile gaming task, the proposed approach reduces setup complexity and cost while preserving discriminative neural information.

### 4.6. Limitations and Future Work

This study has a few limitations that need to be acknowledged. First, the Random Forest model’s overfitting may have been caused by the limited sample size; even though Leave-One-Subject-Out (LOSO) was employed to address this, the findings should be interpreted as exploratory. Second, the research was limited to occipital channels (O1/O2); this approach does not capture frontal executive control or fronto-parietal connectivity associated with inhibitory regulation and decision-making, but it does not show abnormalities in visual processing. To completely map the relationship between “craving” (visual) and “loss of control” (frontal), future research should incorporate the frontal–occipital connection. The relatively small sample size (15 GD and 15 healthy controls) may limit the generalizability and stability of the trained models. Although subject-independent LOSO validation was employed to mitigate overfitting, future studies with larger and more diverse cohorts are necessary to confirm the robustness and clinical applicability of the proposed framework.

The study did not include a non-gaming baseline or an additional control task to dissociate generic visual attention from game-specific engagement. Future work should incorporate baseline or multi-task paradigms to further disentangle task-specific neural signatures.

Furthermore, the use of dry electrodes during a mobile gaming task introduces the possibility of motion-related artifacts in the recorded EEG signals. Although rigorous artifact rejection and filtering were applied, the spectral overlap between movement-related signals (typically <4 Hz) and the delta band means that contributions from micro-movements cannot be fully excluded. Importantly, convergent alterations were also seen in higher-frequency metrics, such as gamma-band signal complexity and beta-band variability, which are less impacted by slow movement but may still be influenced by muscle activity. Together, these findings imply that the observed spectral patterns are unlikely to be explained exclusively by gross motion artifacts. Future work may benefit from the incorporation of automated, learning-based preprocessing techniques, such as CNN-driven decomposition approaches, particularly in higher-density EEG configurations [14].

The results primarily demonstrate the feasibility of combining low-cost, wireless EEG acquisition with machine-learning techniques for behavioral addiction assessment rather than establishing definitive biomarkers. Future studies with larger and more diverse populations are required to validate the robustness and translational applicability of the proposed framework.

An important limitation of the current study is that it does not account for the influence of misinformation and misleading narratives about gaming and behavioral addictions that circulate on digital platforms and social media. Prior research demonstrates that algorithmic amplification on social media can spread false health claims and normalize compulsive gaming behaviors among young users, potentially influencing both genuine gaming patterns and self-reported symptom severity [49,50,51]. Participants’ CSG scores and self-reported gaming hours may have been indirectly shaped by exposure to narratives that minimize gaming disorder risks or misrepresent compulsive engagement as a positive lifestyle choice. Future work should integrate digital health literacy assessment and misinformation detection algorithms with EEG-based biomarker analysis to provide a more comprehensive understanding of how informational environments interact with neurophysiological markers of gaming disorder. Such an interdisciplinary approach would strengthen early detection and intervention by accounting for both neural signatures and the broader digital ecosystem that influences narratives surrounding gaming behavior.

## 5. Conclusions

This work provides novel neurophysiological evidence for the abnormal brain dynamics seen in Gaming Disorder (GD) during active gameplay. By analyzing task-based EEG signals from the occipital visual cortex, we identified a distinct pathological signature in gamers with GD defined by altered spectral power distribution and attentional variability. In contrast to the sustained high-frequency activation observed in healthy controls, the GD group exhibited a dominance of slow-wave activity (Delta/Theta) and suppressed Beta power. This profile suggests that during the addictive act, the GD brain may operate in a state of “neural efficiency”, characterized by automatized processing and reduced cortical effort.

Additionally, our multi-feature analysis identified beta variability and gamma entropy as important biomarkers, indicating a dual disorder of latent sensory desire and unstable top-down attentional control. The relevance of these occipital EEG markers was evaluated using machine learning analysis, in which the Decision Tree classifier achieved the highest generalization performance (accuracy: 80.0%, F1-score: 0.80), demonstrating a proof-of-concept performance in this pilot-scale study. This improved performance demonstrates how interpretable, rule-based algorithms can effectively overcome the overfitting problems seen in high-capacity ensemble models by specifying strong diagnostic criteria for small biomedical datasets.

From an application-oriented perspective, these findings suggest that task-based occipital EEG features may serve as objective neurophysiological indicators for characterizing gaming-related brain processes during active gameplay. Future research should assess the robustness of these EEG features in larger, longitudinal datasets and incorporate frontal–executive connectivity analyses to further elucidate the neural mechanisms underlying gaming-related behavioral dysregulation.

## Figures and Tables

**Table 1:** Artifact Rejection and Signal Quality Metrics (±100 µV Threshold). Values are mean ± SD. Artifact rejection was performed using a fixed peak-to-peak amplitude threshold of ±100 µV. Group comparison was conducted using a two-sided Welch’s *t*-test on rejected seconds. CV denotes the coefficient of variation (SD/mean × 100) and is reported descriptively.

	GD (*n* = 15)	HC (*n* = 15)	Group Comparison
Data Retained (seconds)	496.86 ± 9.25	500.73 ± 14.21	
Data Retained (%)	88.72 ± 1.65	89.42 ± 2.54	
Data Rejected (seconds)	63.14 ± 9.25	59.27 ± 14.21	t (24.1) = 0.88, *p* = 0.385
Data Rejected (%)	11.28 ± 1.65	10.58 ± 2.54	
Coefficient of Variation (rejected seconds, %)	14.66%	23.97%	

**Table 2:** Sensitivity Analysis of Artifact Rejection Across Amplitude Thresholds. Values are mean ± SD. Percentages were computed relative to a fixed analyzed duration of 560 s per subject. *p*-values were obtained using two-sided Welch’s *t*-tests comparing GD and HC rejected seconds at each threshold.

Threshold	Group	Rejected (sec)	Rejected (%)	Retained (%)	Group *p*-Value
±75	GD HC	100.14 ± 16.24 106.34 ± 17.21	17.88 ± 2.90 18.99 ± 3.07	82.12 ± 2.90 81.01 ± 3.07	*p* = 0.322
±100	GD HC	63.14 ± 9.25 59.27 ± 14.21	11.28 ± 1.65 10.58 ± 2.54	88.72 ± 1.65 89.42 ± 2.54	*p* = 0.319
±125	GD HC	37.41 ± 11.04 44.37 ± 13.24	6.68 ± 1.97 7.92 ± 2.36	93.32 ± 1.97 92.08 ± 2.36	*p* = 0.132

**Table 3:** Exploratory group comparisons of occipital EEG features: means, uncorrected *p*-values, and effect sizes (Cohen’s d) for GD and HC gamers.

Feature	Mean (GD)	Mean (HC)	*p*-Value	Cohen’s d
O1_Delta_Activity	4.03 × 10^9^	3.02 × 10^8^	0.067	0.88
O1_Delta_Std	4.20 × 10^4^	1.11 × 10^4^	0.073	0.84
O1_Beta_Complexity	1.13	1.12	0.079	0.80
O2_Theta_Complexity	1.15	1.17	0.088	−0.77
O1_Gamma_Entropy	0.022	0.089	0.110	−0.75

**Table 4:** Sensitivity analysis using non-concatenated (segment-wise) EEG feature extraction. Group comparisons of occipital EEG features computed from artifact-free 10-s segments without temporal concatenation.

Feature	Mean (GD)	Mean (HC)	*p*-Value	Cohen’s d
O1_Delta_Activity	3.97 × 10^9^	3.57 × 10^8^	0.063	0.90
O1_Delta_Std	5.23 × 10^5^	2.14 × 10^4^	0.069	0.92
O1_Beta_Complexity	1.04	1.07	0.089	0.89
O2_Theta_Complexity	1.17	1.14	0.074	−0.89
O1_Gamma_Entropy	0.022	0.089	0.124	−0.51

**Table 5:** Classification performance metrics of five machine learning models in distinguishing GD from HC subjects (*N* = 30). Accuracy is reported as k/n under leave-one-subject-out (LOSO) validation. SEM reflects dispersion of binary per-subject correctness across LOSO folds. Confidence intervals correspond to 95% Wilson score intervals for binomial proportions. Precision, recall, and F1-score are macro-averaged across classes and computed from aggregate LOSO predictions.

Model	Accuracy (k/*n*)	SEM	95% CI (Wilson)	Precision	Recall	F1-Score
Decision Tree	0.800 (24/30)	0.075	[0.63–0.91]	0.800	0.800	0.800
Random Forest	0.533 (16/30)	0.091	[0.36–0.70]	0.526	0.667	0.588
KNN	0.633 (19/30)	0.088	[0.45–0.78]	0.625	0.667	0.645
ANN (MLP)	0.467 (14/30)	0.091	[0.30–0.64]	0.467	0.467	0.467
SVM (RBF)	0.500 (15/30)	0.091	[0.33–0.67]	0.500	0.467	0.483

**Table 6:** Subject-level misclassification patterns and failure modes under LOSO validation. Total errors denote the number of misclassified subjects (out of *n* = 30). Error direction indicates whether GD cases were misclassified as HC (GD → HC) or HC cases were misclassified as GD (HC → GD). The percentage of subjects misclassified summarizes subject-level robustness for each classifier.

Model	Total Errors	GD → HC Errors	HC → GD Errors	% Subjects Misclassified
Decision Tree	6	4	2	20.0%
Random Forest	14	8	6	46.7%
KNN	11	6	5	36.7%
ANN (MLP)	16	9	7	53.3%
SVM (RBF)	15	8	7	50.0%

## Data Availability

The data presented in this study are available from the corresponding authors upon request.
